# Next-generation sequencing of microbial cell-free DNA for rapid noninvasive diagnosis of infectious diseases in immunocompromised hosts

**DOI:** 10.12688/f1000research.19766.4

**Published:** 2020-01-06

**Authors:** Jose F. Camargo, Asim A. Ahmed, Martin S. Lindner, Michele I. Morris, Shweta Anjan, Anthony D. Anderson, Clara E. Prado, Sudeb C. Dalai, Octavio V. Martinez, Krishna V. Komanduri

**Affiliations:** 1 Infectious Diseases, University of Miami Miller School of Medicine, Miami, FL, 33136, USA; 2 Karius, Inc., Redwood City, CA, USA; 3 Department of Pharmacy, University of Miami Sylvester Comprehensive Cancer Center, Miami, FL, 33136, USA; 4 Department of Microbiology, University of Miami Miller School of Medicine, Miami, FL, 33136, USA; 5 Division of Hematology Oncology, University of Miami Miller School of Medicine, Miami, FL, 33136, USA

**Keywords:** Cell-free microbial DNA, next generation sequencing, infection, immunocompromised host, hematopoietic stem cell transplant

## Abstract

**Background:** Cell-free DNA (cfDNA) sequencing has emerged as an effective laboratory method for rapid and noninvasive diagnosis in prenatal screening testing, organ transplant rejection screening, and oncology liquid biopsies but clinical experience for use of this technology in diagnostic evaluation of infections in immunocompromised hosts is limited.

**Methods:** We conducted an exploratory study using next-generation sequencing (NGS) for detection of microbial cfDNA in a cohort of ten immunocompromised patients with febrile neutropenia, pneumonia or intra-abdominal infection.

**Results:** Pathogen identification by cfDNA NGS demonstrated positive agreement with conventional diagnostic laboratory methods in 7 (70%) cases, including patients with proven/probable invasive aspergillosis, *Pneumocystis jirovecii* pneumonia, *Stenotrophomonas maltophilia* bacteremia, Cytomegalovirus and Adenovirus viremia. NGS results were discordant in 3 (30%) cases including two patients with culture negative sepsis who had undergone hematopoietic stem cell transplant in whom cfDNA testing identified the potential etiological agent of sepsis; and one kidney transplant recipient with invasive aspergillosis who had received >6 months of antifungal therapy prior to NGS testing.

**Conclusion:** These observations support the clinical utility of measurement of microbial cfDNA sequencing from peripheral blood for rapid noninvasive diagnosis of infections in immunocompromised hosts. Larger studies are needed.

## Introduction

Infections are a leading cause of morbidity and mortality among immunocompromised individuals ^1– 4^. Bacteremia occurs in up to 25% of all patients with neutropenia and fever ^5^. Infection is a leading cause of non-relapse mortality among hematopoietic cell transplantation (HCT) recipients ^6^. The incidence of bacteremia ^7– 9^ and double-stranded DNA viral reactivation ^10^ is higher than 40% and 90%, respectively, within the first 100 days post-transplant. The cumulative incidence rates of proven/probable invasive fungal infections during the first year after allogeneic HCT with non-myeloablative conditioning is 19% ^11^. Infection is also a common complication of chimeric antigen receptor-modified T (CAR-T)-cell immunotherapy with 28-day cumulative incidence of 23% after CAR-T-cell infusion ^12^.

Establishing a microbiological diagnosis of infectious diseases in this vulnerable population is often challenging for a number of reasons. i) Prior exposure to antibiotics and antifungals which confounds the yield of blood cultures; indeed, most patients with neutropenia and fever will have no infectious etiology documented ^5^. ii) Low sensitivity of mycobacterial and fungal cultures; some microorganisms, such as fastidious bacteria, mycobacteria and dimorphic fungi require longer incubation periods; and blood cultures in almost half of patients with candidemia are negative ^13, 14^. iii) Tissue biopsies are often precluded due to the risk of bleeding in the setting of thrombocytopenia, coagulopathy in those with liver disease or hemodynamic instability in critically ill patients. A delay in diagnosis in patients with invasive fungal infection results in higher mortality ^15, 16^. Thus, there is an unmet need for novel, rapid, cost-effective, noninvasive diagnostic methods in the field.

Cell-free DNA (cfDNA) technology has been used successfully in noninvasive prenatal testing, organ transplant rejection screening, and oncology liquid biopsies ^17– 22^. In recent years, this technology has been developed for use in infectious disease diagnostics ^23, 24^. Detection of microbial cfDNA by next generation sequencing (NGS) is an accurate and precise way of identifying and quantifying pathogens ^25^. The Karius ^®^ Test relies on sequencing of microbial cfDNA circulating in plasma to identify over 1,000 pathogens, including bacteria, viruses and fungi, from a 5 ml blood sample ^25^. This novel diagnostic tool has been recently validated in a study showing that microbial cfDNA NGS identified 94% of microbes identified by conventional blood culture in patients with sepsis ^25^ and has excellent correlation with quantitative PCR testing in patients with cytomegalovirus (CMV) ^23, 25^.

Recent reports indicate that NGS measuring microbial cfDNA is useful in the diagnosis of cases of *Streptococcus pneumoniae-*related hemolytic uremic syndrome, *Coxiella burnetii* endocarditis, invasive *Mycobacterium chimaera* infection, *Nocardia cyriacigeorgica* pneumonia, *Capnocytophaga canimorsus* sepsis, *M. tuberculosis* complex and *M. haemophilum* infections, *M. bovis* aortitis; *Candida* spp., *Aspergillus* spp *.,* non *-Aspergillus* molds invasive infections; *Pneumocystis jirovecii* pneumonia (PJP) *, Toxoplasma gondii* infection and chorioamnionitis, among others ^24, 26– 33^. Among 21 patients with culture-positive infective endocarditis, cfDNA NGS identified the same organism as blood cultures in 20 patients (95% sensitivity) and additionally identified *Enterococcus faecalis* in one out of the three patients with definitive culture-negative endocarditis ^34^. Of note, in this study the cfDNA NGS test identified pathogens causing endocarditis in patients pre-treated with antibiotics up to 30 days prior to initial sample collection.

Here we evaluated the clinical utility of NGS for detection of microbial cfDNA in plasma in a cohort of ten patients receiving chemotherapy or transplants with episodes of febrile neutropenia, sepsis or documented infection.

## Methods

### Study design and study subjects

This was an exploratory study sponsored by Karius, Inc. A total of ten cfDNA kits were provided to the investigators free of charge to be used during a 60-day period. The main goal of this pilot study was to assess the performance of the cfDNA NGS test, compared to standard microbiological evaluation, in immunocompromised patients with documented infection and those undergoing diagnostic evaluation for febrile illness. Patients were enrolled if they had a clinical scenario (e.g., such as fever or pulmonary nodules) suspected or confirmed to be infectious in origin. Half of the patients enrolled in this pilot study had an established diagnosis of infection prior to NGS testing. Our goal in such patients who had documented infection prior to enrollment was to evaluate the positive agreement between NGS and conventional diagnostic testing results. Adult patients followed at the Sylvester Comprehensive Cancer Center were enrolled between July 31 and October 2, 2018. Inclusion criteria were: i) age >18 years old; ii) patients must have received chemotherapy or transplant; and iii) must have had a febrile illness or documented infection (e.g., positive blood cultures, clinical/radiographic evidence of pneumonia). There were no exclusion criteria. The study was approved by the University of Miami Institutional Review Board (IRB approval #20080899), consistent with principles in the Declaration of Helsinki. Each participant provided written informed consent for their inclusion in the study. No sample size calculation was done; instead the number of patients enrolled was entirely dependent on the number of cfDNA kits made available for the pilot study.

### Sample collection and processing

Blood samples (5 mL) were collected in BD vacutainer plasma preparation tubes. Samples were collected at the time of suspected or confirmed infection diagnosis. Within 1 hour of sample collection, tubes were spun down at 1,100 RCF for 10 min at room temperature. Samples were shipped overnight to Karius, Inc. (Redwood City, CA).

### Measurement of cfDNA using NGS

Cell-free DNA was extracted from plasma, NGS libraries were prepared, and sequencing was performed on an Illumina NextSeq ^®^500. Sequencing reads identified as human were removed, and remaining sequences were aligned to a curated pathogen database. Any of over 1,000 organisms in the Karius clinical reportable range found to be present above a predefined statistical threshold were reported as previously described ^24^. The quantity for each organism identified was expressed in Molecules Per Microliter (MPM), the number of DNA sequencing reads from the reported organism present per microliter of plasma.

### The Karius ^®^ Test

***Reference database and QC.*** Reference genomes for *Homo sapiens* and microorganisms (bacteria, viruses, fungi/molds, and other eukaryotic pathogens) were retrieved from the National Center for Biotechnology Information (NCBI) ftp site ( NCBI, U.S. National Library of Medicine (NLM), Human Genome, release GRCh38.p7, and NCBI, U.S. NLM, Microbial Genomes, respectively). Sequence similarities between microorganism references were inspected to identify taxonomic mislabeling and sequence contamination. From the reference genomes passing these quality controls, a subset was selected to maximize sequence diversity. As part of the selection process, NCBI BioSample data were used to ensure the inclusion of reference genomes from both clinical and non-clinical isolates. The final reference genome dataset included over 21,000 reference genomes, containing over 2.7 million sequences. Selected sequences were collected into a single FASTA file and used to generate our microorganism reference BLAST database. A subset of these taxa, including 1251 clinically significant microorganisms, was used as the clinical reportable range.

***Clinical reportable range (CRR).*** The selection of organisms in the clinical reportable range (CRR) was performed as follows. A candidate list was generated by two board-certified infectious disease physicians by including (a) DNA viruses, culturable bacteria, additional fastidious and unculturable bacteria, mycobacteria, and eukaryotic pathogens from a clinical infectious diseases reference textbook ^35^ and a number of infectious disease references, (b) organisms in the pathogen database referenced in published case reports, and (c) reference genomes sequenced from human clinical isolates (as indicated by the NCBI BioSample resource) with publications supporting pathogenicity. Organisms from the above list that were associated with high-quality reference genomes, as determined by our reference database QC process (see above), were used to further narrow the range. Finally, organisms observed as sporadic environmental contamination were excluded from the CRR in order to prevent false-positive calls, e.g., Propionibacterium acnes, Acinetobacter lwoffii, and several Methylobacterium spp. The full list of pathogens detected can be found online: kariusdx.com/pathogenlist/3.3 (where 3.3 is the Karius Test version used in this study). The sequence database is continuously curated to minimize human cross-reactivity as well as cross-reactivity between pathogens and is screened to mitigate contamination with sequences from human or other organisms.

***Sequencing.*** Plasma samples were thawed, centrifuged at 16,000 RCF for 10 min, and spiked with a known concentration of synthetic DNA molecules for quality control purposes. Cell-free DNA was extracted from 0.5 mL plasma using a magnetic bead-based method (Omega Bio-tek Mag-Bind® cfDNA kit; catalog number M3298-01, Norcross, GA). DNA libraries for sequencing are constructed using a modified Ovation® Ultralow System V2 library preparation kit (NuGEN, San Carlos, CA). Negative controls (buffer only instead of plasma) and positive controls (healthy plasma spiked with a known mixture of microbial DNA fragments) were processed alongside patient samples in every batch. Samples were multiplexed with other samples and sequenced on an Illumina NextSeq® 500.

***Analysis pipeline.*** Primary sequencing output files were processed using bcl2fastq (v2.17.1.14) to generate the demultiplexed sequencing reads files. Reads were filtered based on sequencing quality and trimmed based on partial or full adapter sequence. The bowtie2 (version 2.2.4) tool was used to align the remaining reads against Karius’ human and synthetic-molecules references. Sequencing reads exhibiting strong alignment against the human references or the synthetic molecule references were collected and excluded from further analysis. Remaining reads were aligned against Karius’ proprietary microorganism reference database using NCBI-blast (version 2.2.30+). A mixture model was used to assign a likelihood to the complete collection of sequencing reads that included the read sequence probabilities and the (unknown) abundances of each taxon in the sample. An expectation-maximization algorithm was applied to compute the maximum likelihood estimate of each taxon abundance. Only taxa whose abundances rejected the null hypothesis of originating from environmental contamination (as calculated from the negative controls) at high significance levels were reported. The quantity for each organism identified was expressed in molecules per microliter (MPM), the number of DNA sequencing reads from the reported organism present per microliter of plasma. MPM values are calculated from the ratio between the number of sequencing reads assigned to an organism and to an internal control (see Methods in Blauwkamp *et al.* ^25^). Depending on both the concentration of the microbe as well as its genome length, sequencing coverage can range from a few reads and up to >10x for high-concentration shorter viral genomes. Importantly, the MPM value is not affected by sequencing depth or human cell-free DNA concentration in the sample. The entire process from DNA extraction through analysis was typically completed within 28 hours.

## Results

### Background patient information

The characteristics of the patients studied are presented in Table 1. The median age was 56 years (range, 20–65) with 60% of participants being males. Except for a kidney transplant recipient, all other patients had underlying hematological malignancy and/or had received an HCT. All but one (patient #2) were admitted in the hospital at the time of clinical evaluation. All the patients were receiving antimicrobials at the time of plasma sample collection. Three patients had neutropenia (absolute neutrophil count <500/µL) at the time of febrile illness. All febrile patients had blood cultures collected within 24 hours of plasma sample collection for NGS.

**Table 1.** Clinical characteristics of study subjects and results of next-generation sequencing of cell-free DNA.

Patient	Age, gender	Underlying disease	Clinical scenario	Sample from CVC	Days of antibiotics/ antifungals prior to blood draw ^a^	Conventional diagnostic method results ^b^	Microbial cfDNA pathogen results	MPM	Reference values ^c^	Correlation ^d^
1 ^*^	65F	Kidney transplant	Pyogenic intra-abdominal infection	No	18/182	*Aspergillus fumigatus* detected by PCR and culture in abdominal fluid	Negative ( *Aspergillus* *fumigatus* ^e^)	15 ^e^	<10	No ^e^
2	21M	NHL, HCT day +342	Mediastinal lymphadenopathy	No	0/8	Negative fungal serologies and antigens BAL and lymph node tissue cultures negative	Negative			Yes
3 ^*^	20M	AML, HCT day +9	Neutropenic fever, diarrhea	Yes	8/2	CMV detected <137 IU/mL (subsequently peaked at 2,621 IU/mL) Blood cultures and *C. difficile* PCR negative	Cytomegalovirus	108	<10	Yes
4 ^*^	64F	B-ALL MMUD day +291	Fever, cough, lung mass	Yes	6/5	*Pneumocystis jirovecii* BAL PCR+	*Pneumocystis jirovecii*	263	<10	Yes
5	37M	Relapsed DLBCL after CAR-T	Neutropenic fever, weakness, diarrhea, cough	Yes	21/5	Adenovirus 480 copies/mL (subsequently peaked at 2,600 copies/mL)	Adenovirus	845	<10	Yes
6 ^*^	56M	AML, MMUD day +290	Pulmonary nodules (recently diagnosed IA) admitted with SOB	Yes	6/21	CMV detected <137 IU/mL (subsequently peaked at 440 IU/mL) Repeat BAL negative	Cytomegalovirus	93	<10	Yes
7	44M	DLBCL	Fevers, pulmonary nodules	Yes	3/3	Blood cultures negative	*Rothia mucilaginosa*	20	<10	No
8	60F	MDS, HCT day+160, GI-GVHD	Septic shock, multi-organ failure	Yes	15/10	Blood cultures negative	*Escherichia coli* *Lactobacillus* *rhamnosus* Torque teno virus	2,492 308 91	<17 <10 <10	No
9	55F	Multiple myeloma	Pneumonia	Yes	2/0	Negative BAL studies	Negative			Yes
10 ^*f^	58M	AML	Neutropenic fever, pulmonary nodules, sepsis	Yes	120/129	*S. maltophilia* in blood cultures Pan- *Aspergillus* PCR+ in BAL Serum galactomannan+	*Stenotrophomonas* *maltophilia* *Aspergillus oryzae* *Staphylococcus* *epidermidis*	236,594 11,533 9,673	<83 <10 <17	Yes

^*^These patients had documented infection by standard laboratory methods prior to Karius ^®^ Test

^a^ Refers to empiric or targeted therapy only. It does not include days of antimicrobial prophylaxis.

^b^ Blood cultures were obtained within 24h of plasma sample for NGS in all patients and resulted as negative unless specified otherwise in the table.

^c^ Reference value is the 97.5th percentile in self-reported healthy adults for whom the Karius ^®^ Test was performed

^d^Correlation between Karius ^®^ Test and standard laboratory methods

^e^ *Aspergillus fumigatus* reads were present in the raw data but below the statistical threshold for a positive test result. Kidney transplant complicated with perinephric abscess due to *Aspergillus fumigatus* requiring multiple abdominal washouts. The patient had received >6 months of voriconazole and few days of combination therapy with micafungin prior to NGS testing

^f^ Initial cfDNA testing performed 7 weeks prior had only identified *S. epidermidis* and EBV. At that time, BAL and transbronchial biopsy results were unrevealing.

ALL, acute lymphoblastic leukemia; AML, acute myeloid leukemia; BAL, bronchoalveolar lavage; CAR-T, chimeric antigen receptor-modified T-cell immunotherapy; cfDNA, cell-free DNA; CMV, cytomegalovirus; CVC, central venous catheter; DLBCL, diffuse large B cell lymphoma; GI-GVHD, gastrointestinal graft-versus-host disease; HCT, hematopoietic cell transplantation; F, female; M, male; MPM, molecules per microliter; NGS, next-generation sequencing; NLH, Non-Hodgkin lymphoma; SOB, shortness of breath.

### Results of NGS for detecting microbial cfDNA

In this cohort of immunocompromised hosts, pathogen identification by cfDNA NGS demonstrated positive agreement with conventional diagnostic laboratory methods in 7 (70%) cases including positive concordant results in 5 cases and negative concordant results in 2 cases ( Table 1). The kidney transplant recipient had an *Aspergillus fumigatus* perinephric abscess, and *Aspergillus* cfDNA levels, although detected in plasma, were below the positive reporting threshold. However, among patients with hematological malignancy in whom a microbiological diagnosis was established (n=5), cfDNA NGS testing correlated with other methods in all cases. This included patients with proven/probable invasive aspergillosis, PJP, *Stenotrophomonas maltophilia* bacteremia, CMV and adenovirus viremia. Among four patients with hematological malignancy with negative standard laboratory testing, the NGS test identified the potential cause of bacterial sepsis in two patients ( *Rhotia mucilaginosa* in patient #7 and *Escherichia coli* in patient #8; Table 1), both of whom had a compatible clinical scenario and experienced good clinical response to antibiotic therapy with resolution of fever and hypotension.

Five patients (#1, 3, 4, 6, and 10) had documented infection diagnosed by conventional diagnostic methods prior to NGS testing. Notably, the Karius test detected circulating cfDNA of all the organisms identified by conventional diagnostic methods in these five patients, but as mentioned above for patient #1 levels were below the positive reporting threshold ( Table 1). This is, the reported NGS results were concordant with the results of conventional diagnostic laboratory methods in 4 out of 5 patients with documented infection prior to NGS.

After excluding the five patients in whom a microbiological diagnosis was established prior to NGS testing, and the two patients in whom there was negative agreement between conventional testing and NGS (i.e., a diagnosis could not be established), there were only three cases in whom we could assess the impact of NGS results on clinical decision making. In patient #5 adenovirus viremia was detected via NGS, which triggered assessment of adenovirus DNA levels in blood by PCR and ultimately led to initiation of antiviral therapy. In patients #7 and #8 with culture-negative sepsis, NGS did not change management *per se* in terms of escalation or de-escalation of therapy but it supported the diagnosis of bacterial sepsis and both patients completed a course of antibiotic therapy with clinical improvement.

## Discussion

Here we report our experience using cfDNA NGS in the evaluation of immunocompromised patients—predominantly those with hematological malignancy—with febrile illness or documented invasive infections. The study cohort included a heterogeneous group of clinical scenarios, including intra-abdominal infection, pulmonary nodules/pneumonia, neutropenic fever, and septic shock. The results of this proof-of-concept study, where most of the patients had an established diagnosis of infection prior to NGS testing, complement recent reports studying the use of cfDNA NGS in immunocompromised hosts. In a recent study of 55 patients with neutropenic fever, cfDNA testing had positive agreement with conventional blood cultures in 9 of 10 patients in whom blood cultures identified a causative organism of sepsis. Using clinical adjudication by three infectious diseases specialists, cfDNA NGS had a sensitivity of 85.4% (41/48) and specificity of 100% (7/7) ^36^. Thus, this test is a promising diagnostic tool in neutropenic fever, a clinical scenario where conventional work up fails to identify an etiological agent in a majority of cases ^5^. Another study evaluated 40 pediatric patients with prolonged neutropenia and fever (>96h) despite administration of antibiotics for suspected fungal infection (the authors excluded patients who had received antifungal therapy for >4 days); in this study cfDNA NGS identified fungal pathogens including *Aspergillus fumigatus*, *Rhizopus* spp., *Candida albicans*, *Candida glabrata* and *Pneumocystis jirovecii* ^37^.

Except for patients diagnosed with viral infections (e.g., patients #3, #5 and #6 with adenovirus or CMV viremia), all other patients were receiving antimicrobial therapies that were active against the organism(s) identified ( Table 1) suggesting that NGS may be able to detect organisms in the setting of effective treatment. For example, patient #4 who was diagnosed with PJP, had detectable levels of *Pneumocystis jirovecii* DNA in blood despite receiving three days of trimethoprim/sulfamethoxazole treatment dose at the time of NGS testing; and patient #10 had positive NGS testing for *Aspergillus oryzae* despite having received >120 days of anti-mold therapy including triple antifungal regimen (isavuconazole, micafungin and liposomal amphotericin B) at the time of NGS testing.

There is limited data on Karius test performance for invasive mold infections. In a retrospective case-control study of 57 HCT recipients with proven/probable pulmonary invasive mold infections, the cfDNA NGS test identified 83% (5/6) of molds among patients with non- *Aspergillus* infections; but among those with *Aspergillus* proven/probable disease, *Aspergillus fumigatus* was only identified in 13.7% (7/51) of cases ^38^. In the report by Armstrong *et al*. ^37^, in a cohort of 40 pediatric hematology-oncology and HCT patients, sequencing of circulating cfDNA detected fungal pathogens in five of seven cases with proven and probable invasive fungal disease, and correlated with microbiological diagnosis in four of six proven cases. In a recent report by Hong *et al.* ^24^, in seven out of nine subjects (including seven immunocompromised hosts) with proven invasive fungal infection, plasma NGS testing detected the same fungus identified from the biopsy tissue at the genus level. The fungi identified by plasma NGS included *Aspergillus* spp. and non- *Aspergillus* molds such as *Scedosporium*, *Rhizopus*, and *Cunninghamella* ^24^. In that report, there was one case where the plasma sample was obtained after at least 15 days of anti- *Aspergillus* therapy, and NGS testing did not identify the causal organism of invasive fungal infection. Similarly, for the kidney transplant patient reported here with invasive aspergillosis, in whom *Aspergillus fumigatus* cfDNA levels in plasma were detected below the reporting threshold, six months of anti- *Aspergillus* therapy (including combination of voriconazole plus micafungin at the time of NGS testing) had been administered prior to the time of plasma collection. Thus, prolonged antifungal therapy prior to sample collection (e.g., >7–14 days) might interfere with detection of fungal DNA. One exception to this might be patients with profound prolonged neutropenia (e.g., absolute neutrophil count <100 cells/mL for more than 7 days) and those with refractory acute leukemia, in whom NGS might detect *Aspergillus spp*. DNA in peripheral blood despite significant exposure to antifungal therapy like it occurred with patient #10.

Although NGS has been used for screening of allograft rejection in solid organ transplant recipients ^17– 19, 23^, there are limited data with the use of NGS for diagnosis of infections in this population. A recent study demonstrated strong correlation between clinical test results and cfDNA derived from CMV in a cohort of lung transplant recipients ^23^. In addition, cfDNA revealed undiagnosed cases of infection with microsporidia and pathogenic viruses, including adenovirus and human herpesvirus 6 among lung transplant patients ^23^.

Recently, Fung *et al*. reported three patients who received allogeneic HCT transplant in whom NGS cfDNA facilitated the diagnosis of an uncommon presentation of *Chlamydia trachomatis* and recurrent and metastatic complications of *Staphylococcus aureus* bacteremia before standard microbiology ^39^.

The fact that in our cohort cfDNA NGS testing identified the potential cause of febrile illness in two patients with culture-negative sepsis who had a compatible clinical syndrome and responded well to antibiotic therapy supports the notion that NGS testing can be a useful diagnostic tool, particularly when conventional blood cultures are negative. The Karius ^®^ Test pathogen-specific reference ranges have been established using cfDNA levels from healthy donors. Patient #8 had detectable levels of Torque teno virus, which belongs to *Anelloviridae* family and is considered to lack pathogenic potential; this patient also had detection of *Lactobacillus* spp, which is part of normal gastrointestinal flora and usually interpreted as a contaminant when isolated from blood cultures. Similarly, for patient #10, in addition to pathogenic organisms such as *Aspergillus* sp. and *Stenotrophomonas maltophilia*, cfDNA of *Staphylococcus epidermidis*, of unclear clinical significance in this patient and likely contaminant, was also detected. This suggests the possibility that cfDNA NSG might on occasion yield detection of members of the commensal microbiota or viroma. Thus, the results of cfDNA NGS technology need to be interpreted with caution and in conjunction with other laboratory, radiological and clinical findings.

To our surprise, however, even though many of the patients tested had mucosal barrier damage (e.g., mucositis) allowing for bacterial translocation from the gut, the Karius ^®^ Test did not show a non-specific gut flora signal. The test was negative in patients in whom we failed to establish a microbiological diagnosis for their febrile illness, and when positive, typically correlated with conventional laboratory testing. Whether the currently defined cfDNA thresholds are optimal for identifying and quantifying pathogens of clinical relevance in highly vulnerable immunocompromised hosts will require further study. Importantly, the turnaround time for results was consistently within 48 hours, which is quite rapid considering that samples were shipped overnight from our institution located in Florida to the Karius Inc. laboratory in California.

Lack of control group, small number of patients and the heterogeneity of the cohort in terms of underlying diseases and causes of immunosuppression represent major limitations of this report. Larger clinical trials evaluating plasma NGS in patients with cancer and undergoing transplant are ongoing ( NCT03226158, NCT03262584, NCT02912117, NCT02804464). Until larger cohort data becomes available, our observations suggest that detection of microbial cfDNA using NGS is valuable for the rapid noninvasive diagnosis of infectious complications following chemotherapy or transplantation.

## Conclusion

In this small cohort of immunocompromised hosts, the NGS correlated with standard microbiological testing in 70% of cases suggesting this technology might be useful in this clinical setting, particularly for patients in whom bronchoscopy or biopsy for tissue diagnosis is not feasible. As with other novel laboratory diagnostics used in clinical practice, the results of cfDNA NGS technology need to be interpreted with caution and in conjunction with other laboratory, radiological and clinical findings. Larger studies are needed to validate these findings.

## Data availability

### Underlying data

Microbial cfDNA NGS for Rapid Noninvasive Diagnosis of Infectious Diseases in Immunocompromised Hosts, BioProject accession number PRJNA554271

## References

[1] Tomblyn M Chiller T Einsele H: Guidelines for preventing infectious complications among hematopoietic cell transplantation recipients: a global perspective. *Biol Blood Marrow Transplant.* 2009;15(10):1143–1238. 10.1016/j.bbmt.2009.06.019 19747629 PMC3103296

[2] Fishman JA: Infection in solid-organ transplant recipients. *N Engl J Med.* 2007;357(25):2601–2614. 10.1056/NEJMra064928 18094380

[3] Syrjala H Ohtonen P Kinnunen U: Blood stream infections during chemotherapy-induced neutropenia in adult patients with acute myeloid leukemia: treatment cycle matters. *Eur J Clin Microbiol Infect Dis.* 2010;29(10):1211–1218. 10.1007/s10096-010-0984-1 20556469

[4] Ballen K Woo Ahn K Chen M: Infection Rates among Acute Leukemia Patients Receiving Alternative Donor Hematopoietic Cell Transplantation. *Biol Blood Marrow Transplant.* 2016;22(9):1636–1645. 10.1016/j.bbmt.2016.06.012 27343716 PMC5008458

[5] Freifeld AG Bow EJ Sepkowitz KA: Clinical practice guideline for the use of antimicrobial agents in neutropenic patients with cancer: 2010 update by the infectious diseases society of america. *Clin Infect Dis.* 2011;52(4):e56–93. 10.1093/cid/cir073 21258094

[6] Yoo JH Lee DG Choi SM: Infectious complications and outcomes after allogeneic hematopoietic stem cell transplantation in Korea. *Bone Marrow Transplant.* 2004;34(6):497–504. 10.1038/sj.bmt.1704636 15286689

[7] Bock AM Cao Q Ferrieri P: Bacteremia in blood or marrow transplantation patients: clinical risk factors for infection and emerging antibiotic resistance. *Biol Blood Marrow Transplant.* 2013;19(1):102–108. 10.1016/j.bbmt.2012.08.016 22940054

[8] Kikuchi M Akahoshi Y Nakano H: Risk factors for pre- and post-engraftment bloodstream infections after allogeneic hematopoietic stem cell transplantation. *Transpl Infect Dis.* 2015;17(1):56–65. 10.1111/tid.12345 25580541

[9] Gudiol C Garcia-Vidal C Arnan M: Etiology, clinical features and outcomes of pre-engraftment and post-engraftment bloodstream infection in hematopoietic SCT recipients. *Bone Marrow Transplant.* 2014;49(6):824–830. 10.1038/bmt.2014.37 24662420

[10] Hill JA Mayer BT Xie H: The cumulative burden of double-stranded DNA virus detection after allogeneic HCT is associated with increased mortality. *Blood.* 2017;129(16):2316–2325. 10.1182/blood-2016-10-748426 28209721 PMC5399484

[11] Fukuda T Boeckh M Carter RA: Risks and outcomes of invasive fungal infections in recipients of allogeneic hematopoietic stem cell transplants after nonmyeloablative conditioning. *Blood.* 2003;102(3):827–833. 10.1182/blood-2003-02-0456 12689933

[12] Hill JA Li D Hay KA: Infectious complications of CD19-targeted chimeric antigen receptor-modified T-cell immunotherapy. *Blood.* 2018;131(1):121–130. 10.1182/blood-2017-07-793760 29038338 PMC5755046

[13] Clancy CJ Nguyen MH: Finding the "missing 50%" of invasive candidiasis: how nonculture diagnostics will improve understanding of disease spectrum and transform patient care. *Clin Infect Dis.* 2013;56(9):1284–1292. 10.1093/cid/cit006 23315320

[14] Miller JM Binnicker MJ Campbell S: A Guide to Utilization of the Microbiology Laboratory for Diagnosis of Infectious Diseases: 2018 Update by the Infectious Diseases Society of America and the American Society for Microbiology. *Clin Infect Dis.* 2018;67(6):813–816. 10.1093/cid/ciy584 30169655

[15] Morrell M Fraser VJ Kollef MH: Delaying the empiric treatment of *candida* bloodstream infection until positive blood culture results are obtained: a potential risk factor for hospital mortality. *Antimicrob Agents Chemother.* 2005;49(9):3640–3645. 10.1128/AAC.49.9.3640-3645.2005 16127033 PMC1195428

[16] von Eiff M Roos N Schulten R: Pulmonary aspergillosis: early diagnosis improves survival. *Respiration.* 1995;62(6):341–347. 10.1159/000196477 8552866

[17] Bloom RD Bromberg JS Poggio ED: Cell-Free DNA and Active Rejection in Kidney Allografts. *J Am Soc Nephrol.* 2017;28(7):2221–2232. 10.1681/ASN.2016091034 28280140 PMC5491290

[18] Schutz E Fischer A Beck J: Graft-derived cell-free DNA, a noninvasive early rejection and graft damage marker in liver transplantation: A prospective, observational, multicenter cohort study. *PLoS Med.* 2017;14(4):e1002286. 10.1371/journal.pmed.1002286 28441386 PMC5404754

[19] De Vlaminck I Valantine HA Snyder TM: Circulating cell-free DNA enables noninvasive diagnosis of heart transplant rejection. *Sci Transl Med.* 2014;6(241):241ra277. 10.1126/scitranslmed.3007803 24944192 PMC4326260

[20] Allyse MA Wick MJ: Noninvasive Prenatal Genetic Screening Using Cell-free DNA. *JAMA.* 2018;320(6):591–592. 10.1001/jama.2018.9418 30073283

[21] Rossi G Mu Z Rademaker AW: Cell-Free DNA and Circulating Tumor Cells: Comprehensive Liquid Biopsy Analysis in Advanced Breast Cancer. *Clin Cancer Res.* 2018;24(3):560–568. 10.1158/1078-0432.CCR-17-2092 29180605

[22] Ulrich BC Paweletz CP: Cell-Free DNA in Oncology: Gearing up for Clinic. *Ann Lab Med.* 2018;38(1):1–8. 10.3343/alm.2018.38.1.1 29071812 PMC5700141

[23] De Vlaminck I Martin L Kertesz M: Noninvasive monitoring of infection and rejection after lung transplantation. *Proc Natl Acad Sci U S A.* 2015;112(43):13336–13341. 10.1073/pnas.1517494112 26460048 PMC4629384

[24] Hong DK Blauwkamp TA Kertesz M: Liquid biopsy for infectious diseases: sequencing of cell-free plasma to detect pathogen DNA in patients with invasive fungal disease. *Diagn Microbiol Infect Dis.* 2018;92(3):210–213. 10.1016/j.diagmicrobio.2018.06.009 30017314

[25] Blauwkamp TA Thair SA Rosen MJ: Analytical and clinical validation of a microbial cell-free DNA sequencing test for infectious disease. *Nat Microbiol.* 2019;4(4):663–674. 10.1038/s41564-018-0349-6 30742071

[26] Yonts A Farnaes L Venkatasubrahmanyam S: *Streptococcus pneumoniae*-Related Hemolytic Uremic Syndrome (pHUS) and the Identification of Matched Cross-Country Strains by Next-Generation Sequencing (NGS). IDWeek.2018.

[27] Kondo MD Dalai S Westblade L: Diagnosis and Genotyping of *Coxiella burnetii* Causing Endocarditis in a Patient with Prosthetic Pulmonary Valve Replacement (PVR) Using Next-Generation Sequencing (NGS) of Plasma. *Open Forum Infect Dis.*aper presented at: IDWeek,2018;5(suppl_1):S11–S12. 10.1093/ofid/ofy209.024 PMC6580995 31249846

[28] Nayakwadi-Singer M Johnston S Kulhanjian J: Detection of Nocardia cyriacigeorgica from a Deep Pulmonary Infection Using a Novel Plasma-Based Next Generation Sequencing Assay. *ASM Microbe.* 2017.

[29] Nomura J Rieg G Bluestone G: Rapid Detection of Invasive *Mycobacterium chimaera* Infection by Using a Novel Plasma-Based Next-Generation Sequencing Assay. *Open Forum Infect Dis.* 2017;4(suppl_1):S174 10.1093/ofid/ofx163.315 PMC6498503 31046692

[30] Strand A Hong DK Fowler VG Jr: Diagnosis of Capnocytophaga canimorsus Sepsis by a Novel Cell-free DNA Sequencing Assay. *ASM Microbe.* 2016.

[31] Hong DK Truong C N B: Diagnosis of Mycobacterium tuberculosis and Non-tuberculous Mycobacterium Infections Using a Novel Plasma-Based Next-Generation Sequencing Assay. *ASM Microbe* 2016.

[32] Cooper C Hong D Blair L: Diagnosis of BCG Aortitis by Plasma Metagenomic Sequencing. *Open Forum Infect Dis.*IDWeek;2018;5(suppl_1):S290 10.1093/ofid/ofy210.817

[33] Witt RG Blair L Frascoli M: Detection of Pathogen Cell-Free DNA in Maternal Plasma in Patients with Chorioamnionitis Using a Non-Invasive Next-Generation Sequencing Assay. *SRI 65th Annual Scientific Meeting.* 2018.

[34] Shah P Ruffin F Seng H: Direct Detection and Quantification of Bacterial Cell-free DNA in Patients with Infective Endocarditis (IE) Using the Karius Plasma Next Generation Sequencing (NGS) Test. *Open Forum Infect Dis.*IDWeek;2018;5(suppl_1):S12 10.1093/ofid/ofy209.026

[35] Bennett JE Dolin R Blaser MJ: Mandell, Douglas, and Bennett's Principles and Practice of Infectious Diseases.(Saunders, Philadelphia, PA;2014). Reference Source

[36] Benamu E Gajurel K Anderson JN: Evaluation of the Karius Plasma Next-Generation Sequencing Cell-free Pathogen DNA Test to Determine the Etiology of Infection and Impact on Anti-Microbial Management in Patients with Severe Neutropenia and Fever. *Open Forum Infect Dis.*IDWeek;2018;5(Suppl 1):S680 10.1093/ofid/ofy210.1947

[37] Armstrong A Rossoff J Hollemon D: Cell-free DNA next-generation sequencing successfully detects infectious pathogens in pediatric oncology and hematopoietic stem cell transplant patients at risk for invasive fungal disease. *Pediatr Blood Cancer.* 2019;66(7):e27734. 10.1002/pbc.27734 30941906

[38] Hill JA Hong DK Dalai SC: Retrospective Case-Control Study of the Performance of Cell-Free Plasma Sequencing to Detect Invasive Mold Infections in Hematopoietic Cell Transplant Recipients with Pneumonia. *Biol Blood Marrow Transplant.* 2019;25(3 Supplement):S365–S366. 10.1016/j.bbmt.2018.12.592

[39] Fung M Zompi S Seng H: Plasma Cell-Free DNA Next-Generation Sequencing to Diagnose and Monitor Infections in Allogeneic Hematopoietic Stem Cell Transplant Patients. *Open Forum Infect Dis.* 2018;5(12):ofy301. 10.1093/ofid/ofy301 30581881 PMC6297859

