# Supplementary material for: Next-generation sequencing of microbial cell-free DNA for rapid noninvasive diagnosis of infectious diseases in immunocompromised hosts
Source: F1000Res. 2020 Jan 6;8:1194. Originally published 2019 Jul 26. [Version 4] doi: 10.12688/f1000research.19766.4 (PMC6883395; doi:10.12688/f1000research.19766.4)
Supplement: Peer review history

# Peer review: Reviewer response for version 4

**DOI:** 10.5256/f1000research.24150.r58240

**Published:** 2020-01-22

**Authors:** Steve Miller

**Affiliations:** 1 Department of Laboratory Medicine, University of California, San Francisco, San Francisco, CA, USA

I will approve version 4 of the article.

I confirm that I have read this submission and believe that I have an appropriate level of expertise to confirm that it is of an acceptable scientific standard.

# Peer review: Reviewer response for version 3

**DOI:** 10.5256/f1000research.23607.r56885

**Published:** 2019-11-28

**Authors:** Dimitrios Farmakiotis

**Affiliations:** 1 Transplant and Oncology Infectious Diseases, Warren Alpert Medical School, Brown University, Providence, RI, USA

My queries have been adequately addressed, I have no further comments.

I confirm that I have read this submission and believe that I have an appropriate level of expertise to confirm that it is of an acceptable scientific standard.

# Peer review: Reviewer response for version 3

**DOI:** 10.5256/f1000research.23607.r56887

**Published:** 2019-11-27

**Authors:** Steve Miller

**Affiliations:** 1 Department of Laboratory Medicine, University of California, San Francisco, San Francisco, CA, USA

This submission (v3) by Camargo *et al.* describes the use of plasma cell-free DNA metagenomic sequencing (cf-mNGS) for pathogen detection performed by Karius in a limited number of immunocompromised patients. Results showed that the cf-mNGS results detected the same organism as conventional microbiological methods in 5 patients (one patient had two organisms detected by both conventional and cf-mNGS methods). One patient with a known diagnosis of *Aspergillus fumigatus* had negative cf-mNGS results, although organism reads were detected below reporting thresholds. There were four patients with no conventional diagnosis; two of these had negative cf-mNGS results, and 2 had additional organisms detected by cf-mNGS. There were 5 additional organisms detected by cf-mNGS in 3 patients, of these 2 were adjudicated as clinically significant (not stated which in paper, but I’m presuming *E. coli* and *R. mucilaginosa*) that may have caused clinical sepsis, as the patients responded to therapy which covered these species. In addition to these organisms adjudicated as clinically significant, cf-mNGS detected 3 organisms in 2 patients that were clinically adjudicated to be not significant.

 The paper is generally well written and understandable, although the small number of patients enrolled limits the conclusions that can be drawn, and it would be useful to see the authors expand the study group. For the study design, it is particularly important to know whether these patients were enrolled due to their interesting conventional results and clinical course or whether all patients admitted during this time frame were enrolled. This sort of clinical trial is highly susceptible to bias due to “cherry-picking” patients with severe infections that are highly likely to yield positive cf-mNGS findings.

 Most of the clinical need for diagnostic utility comes in patients that are undiagnosed at the time testing is sent (often despite extensive workup), and the test performance in this population may be very different. Most of the patients had an established diagnosis prior to sending cf-mNGS, and it would be best to show in the tables whether the diagnosis was already known at the time cf-mNGS testing was sent.

 This version improves on the prior description of cf-mNGS test sensitivity, but the concordant results should be broken down into positive concordant results (5 cases) and negative concordant results (2 cases), along with additional organism detections.

 The statement made in the abstract and throughout the article that cf-mNGS identified the etiological agent in two patients with culture negative sepsis is misleading and overly interpreted by the authors. The data show that cf-mNGS detected 5 additional organisms in 3 patients. Of these, two were clinically interpreted as potentially consistent with the patient’s suspected infection. I don’t think the article actually says which cases, but from the table I’m presuming that they intend the *E. coli* and Rothia findings. However, without additional discrepancy testing, one cannot say that this has made the diagnosis. It does seem that the *E. coli* finding with high MPM is likely clinically significant. The case with *Rothia mucilaginosa* is more suspect, it was detected with relatively low MPM, and Rothia is a reasonably common contaminant organism that can be seen in mNGS data.

 The authors don’t discuss how certain organisms were interpreted to be clinically insignificant other than TTV which is well established as a non-pathogen. Presumably the *Lactobacillus rhamnosus* was thought to be a likely contaminant. This is a reasonable clinical interpretation, but again is not definitive. The finding of *Staphylococcus epidermidis* is even more problematic as this can certainly cause clinical sepsis, and it was found at fairly high MPM. It could also be a sample contaminant, and so is very difficult to clinically adjudicate. Unfortunately, clinical response while on therapy that should cover the organism is still not proof that the organism caused the infection or was a true positive finding. In order to make a definitive statement about diagnosis, additional discrepancy testing via orthogonal methods would be required.

 In the discussion, the authors state sensitivity and specificity after clinical adjudication, and also state that the cfDNA NGS testing “identified the cause of febrile illness in two patients with culture-negative sepsis”, which is not accurate as described above. Without true gold standard results, sensitivity and specificity are not the appropriate terms. Given the issues with clinical interpretation, the authors should provide positive and negative agreement before and after clinical adjudication, and the reasons for their interpretations should be shown. The detection of additional organisms that were not found on other tests is a potential strength of cf-mNGS testing, but is also very difficult to interpret, and there is potential for many false positives. Clinical interpretation in these cases is important, and the authors should provide more detail on their approach to this and how to deal with potential false-positive results.

 The interpretation of cf-mNGS test utility needs to be done using objective standards, and requires a prospective study design (without inclusion of cases with known diagnosis). The authors should show whether patients were already on empiric therapy that covered the organism detected or not, and whether any change was made to therapy decisions based on results, along with clinical outcomes of therapy. This ability (or at least potential) to modify treatment decisions based on cf-mNGS is an important outcome metric, especially since definitive outcome studies (clinical cure) require large numbers of patients to show effectiveness. However, if the cf-mNGS testing was sent retrospectively or on known diagnosis cases already under treatment, that should be stated for these cases since there is not a potential to change therapy in those situations. Essentially the reader will want to know how test results changed patient management, which is not shown here.

 Patient #3 (CMV viremia) seems excluded from the discussion of viral infection in the second paragraph of the discussion.

 The *Aspergillus fumigatus* case with reads detected below thresholds is interesting and could have more discussion about whether this finding would be communicated to providers for potential followup testing or correlation with other data. The presentation in Table 1 is confusing as the MPM is shown as 15 with reference value < 10, seemingly a positive result above threshold, but the text states that it is below threshold.

 The technical description of the Karius test is fairly general, which is understandable given that it is proprietary, but it does not allow other researchers to assess it independently. More detail could be provided regarding the thresholds for reporting. For example, in the Analysis Pipeline section, the actual quantitation for “high significance” needed to report organisms that are not environmental contaminants as shown by negative controls should be shown. Adequate sequencing depth should be stated as well as the number of base pairs sequenced. The selection of organisms for reporting and organisms excluded from reporting should be referenced or potentially shown in an appendix.

 The methods state that MPM value is not affected by human cell-free DNA concentration, but others have shown that the human DNA portion does affect the number of reads recovered. The authors should state of reference how normalization is done to adjust for samples with differing amounts of human cell-free DNA.

I confirm that I have read this submission and believe that I have an appropriate level of expertise to confirm that it is of an acceptable scientific standard, however I have significant reservations, as outlined above.

# Response

**Published:** 2019-12-23

**Authors:** Jose Camargo

**Affiliations:** University of Miami Miller School of Medicine, USA

This submission (v3) by Camargo et al. describes the use of plasma cell-free DNA metagenomic sequencing (cf-mNGS) for pathogen detection performed by Karius in a limited number of immunocompromised patients. Results showed that the cf-mNGS results detected the same organism as conventional microbiological methods in 5 patients (one patient had two organisms detected by both conventional and cf-mNGS methods). One patient with a known diagnosis of Aspergillus fumigatus had negative cf-mNGS results, although organism reads were detected below reporting thresholds. There were four patients with no conventional diagnosis; two of these had negative cf-mNGS results, and 2 had additional organisms detected by cf-mNGS. There were 5 additional organisms detected by cf-mNGS in 3 patients, of these 2 were adjudicated as clinically significant (not stated which in paper, but I’m presuming E. coli and R. mucilaginosa) that may have caused clinical sepsis, as the patients responded to therapy which covered these species. In addition to these organisms adjudicated as clinically significant, cf-mNGS detected 3 organisms in 2 patients that were clinically adjudicated to be not significant.

**Authors’ response: we thank the reviewer for his insightful comments/suggestions that have improved the quality of the manuscript.**

 The paper is generally well written and understandable, although the small number of patients enrolled limits the conclusions that can be drawn, and it would be useful to see the authors expand the study group. For the study design, it is particularly important to know whether these patients were enrolled due to their interesting conventional results and clinical course or whether all patients admitted during this time frame were enrolled. This sort of clinical trial is highly susceptible to bias due to “cherry-picking” patients with severe infections that are highly likely to yield positive cf-mNGS findings.

**Authors’ response:  We have edited the methods and results to improve clarity.**

**We agree with the reviewer that the small cohort size is a limitation and for this reason we acknowledge that this data should be considered preliminary and results validated in larger cohorts. However, we feel our observations suggest potential clinical utility of NGS in immunocompromised hosts with febrile illness, particularly those in whom invasive diagnostic methods (e.g. tissue biopsy) are precluded, and as such these observations set the foundation for larger studies.**

**With regards to potential selection bias, this pilot project was designed as proof-of-concept study to assess the performance of the NGS in the “real-world” clinical setting and the agreement rate with conventional diagnostic testing.  The Karius kits were provided to the investigators free of charge for a 60-day period so efforts were made to use all the kits prior to such deadline. As such, the patient population was not completely homogenous. Patients were enrolled if they had a clinical scenario (e.g, fever, pulmonary nodules)  suspected or confirmed to be infectious in origin, and indeed most (4/7) patients in whom Karius was positive (e.g., such as patient #4 with PJP and patient #10 with invasive aspergillosis) had documented infection prior to Karius testing. Our goal in such patients who had documented infection was to evaluate the positive agreement between NGS and conventional diagnostic testing results.   The other three patients correspond to the two cases of culture negative sepsis where Karius identified etiological agent of septicemia (patients #7 and #8), and one patient (patient #5) in whom Adenovirus (AdV) viremia detected via NGS triggered serial assessment of AdV DNA levels by PCR and ultimately led to initiation of antiviral therapy.  In patient #1 diagnosis of invasive aspergillosis was known prior to Karius testing but NGS was negative, likely due to chronic antifungal therapy prior to NGS negatively impacting yield of the test.  Five patients with no documented infection at the time of NGS, underwent NGS testing concurrent with standard microbiological evaluation for febrile illness which is often due to infection in immunocompromised hosts, and so clearly –as stated by reviewer- pre-test probability of a positive result was high. As stated in the conclusion of the manuscript, larger cohort studies are needed.**

 Most of the clinical need for diagnostic utility comes in patients that are undiagnosed at the time testing is sent (often despite extensive workup), and the test performance in this population may be very different. Most of the patients had an established diagnosis prior to sending cf-mNGS, and it would be best to show in the tables whether the diagnosis was already known at the time cf-mNGS testing was sent.

**Authors’ response:  We agree with reviewer. cf-mNGS is most useful in patients in whom previous work up has been non diagnostic and those in whom invasive procedures such as bronchoscopy or tissue biopsy are precluded due to low cell counts or hemodynamic instability. It is in these settings where cf-mNGS can provide information that conventional testing would not.  Thus, your point is well taken and we have added information regarding whether the diagnosis was already known at the time cf-mNGS testing in the revised methods, results and Table.**

 This version improves on the prior description of cf-mNGS test sensitivity, but the concordant results should be broken down into positive concordant results (5 cases) and negative concordant results (2 cases), along with additional organism detections.

**Authors’ response:  This break down of positive and negative concordant results has been added to the revised version. Specific organism detections are outlined in Table 1.**

 The statement made in the abstract and throughout the article that cf-mNGS identified the etiological agent in two patients with culture negative sepsis is misleading and overly interpreted by the authors. The data show that cf-mNGS detected 5 additional organisms in 3 patients. Of these, two were clinically interpreted as potentially consistent with the patient’s suspected infection. I don’t think the article actually says which cases, but from the table I’m presuming that they intend the E. coli and Rothia findings. However, without additional discrepancy testing, one cannot say that this has made the diagnosis. It does seem that the E. coli finding with high MPM is likely clinically significant. The case with Rothia mucilaginosa is more suspect, it was detected with relatively low MPM, and Rothia is a reasonably common contaminant organism that can be seen in mNGS data.

**Authors’ response:  We have edited manuscript (both abstract and results section) to avoid strong statements and emphasize suspected culprit of sepsis in these two patients. For patient #8 *E coli* is one of the more commonly isolated organisms in HCT recipients with bacteremia (Kikuchi et al, Transpl Infect Dis.2015;17(1):56-65;  Gudiol et al, Bone Marrow Transplant. 2014; 49(6): 824–830), and it was likely the cause of sepsis; although since GI source was suspected, polymicrobial infection with other organisms detected such as *Lactobacillus* is also a possibility.  We agree that low MPM for *Rhotia* in patient #7 it is more difficult to interpret but *Rhotia* is also known to cause serious infections and bacteremia in transplant and cancer patients (Ramanan et al, J Clin Microbiol. 2014 Sep;52(9):3184-9. doi: 10.1128/JCM.01270-14; Abidi et al,** **Diagn Microbiol Infect Dis. 2016 May;85(1):116-20.)**

 The authors don’t discuss how certain organisms were interpreted to be clinically insignificant other than TTV which is well established as a non-pathogen. Presumably the Lactobacillus rhamnosus was thought to be a likely contaminant. This is a reasonable clinical interpretation, but again is not definitive. The finding of Staphylococcus epidermidis is even more problematic as this can certainly cause clinical sepsis, and it was found at fairly high MPM. It could also be a sample contaminant, and so is very difficult to clinically adjudicate. Unfortunately, clinical response while on therapy that should cover the organism is still not proof that the organism caused the infection or was a true positive finding. In order to make a definitive statement about diagnosis, additional discrepancy testing via orthogonal methods would be required.

**Authors’ response: As mentioned above it is difficult to establish if *Lactobacillus *was playing a pathogenic role as part of polymicrobial infection in the setting of sepsis of GI source (this patient had severe GI GVHD).  In our opinion, detection of *Staphylococcus epidermidis* most likely had no clinical significance based on the clinical picture where fever and pulmonary nodules were likely related to *Aspergillus* and septic shock occurred in the setting of *S. maltophilia* bacteremia. This has been added to the discussion.**

 In the discussion, the authors state sensitivity and specificity after clinical adjudication, and also state that the cfDNA NGS testing “identified the cause of febrile illness in two patients with culture-negative sepsis”, which is not accurate as described above. Without true gold standard results, sensitivity and specificity are not the appropriate terms. Given the issues with clinical interpretation, the authors should provide positive and negative agreement before and after clinical adjudication, and the reasons for their interpretations should be shown. The detection of additional organisms that were not found on other tests is a potential strength of cf-mNGS testing, but is also very difficult to interpret, and there is potential for many false positives. Clinical interpretation in these cases is important, and the authors should provide more detail on their approach to this and how to deal with potential false-positive results.

**Authors’ response: We have edited manuscript to avoid strong statements. We also edited the discussion to emphasize that detection of commensal organisms can occur and results need to be interpreted with caution and in the context of other clinical, laboratory and radiological findings.**

 The interpretation of cf-mNGS test utility needs to be done using objective standards, and requires a prospective study design (without inclusion of cases with known diagnosis). The authors should show whether patients were already on empiric therapy that covered the organism detected or not, and whether any change was made to therapy decisions based on results, along with clinical outcomes of therapy. This ability (or at least potential) to modify treatment decisions based on cf-mNGS is an important outcome metric, especially since definitive outcome studies (clinical cure) require large numbers of patients to show effectiveness. However, if the cf-mNGS testing was sent retrospectively or on known diagnosis cases already under treatment, that should be stated for these cases since there is not a potential to change therapy in those situations. Essentially the reader will want to know how test results changed patient management, which is not shown here.

**Authors’ response:  Table 1 includes the days of antimicrobial (empiric and targeted, not prophylaxis) that each patient received prior to blood sample collection for NSG, and in the second paragraph of the discussion we mention that “except for patients diagnosed with viral infections, all other patients were receiving antimicrobial therapies that were active against the organism(s) identified”.**

**Given the limitations with study design, after excluding the five patients in whom a diagnosis was known prior to NGS testing, and the two patients in whom both conventional testing and NGS were negative, there are only three cases in whom we could assess modifications in treatment decisions based on NGS.   In patient #5 Adenovirus (AdV) viremia was detected via NGS which triggered serial assessment of AdV DNA levels by PCR and ultimately led to initiation of antiviral therapy. In patients #7 and 8 with culture-negative sepsis, NGS did not change management per se in terms of escalation or de-escalation of therapy but it supported the diagnosis of bacterial sepsis and both patients completed courses of antibiotic therapy with clinical improvement.  This information has been added to the discussion in the revised version.**

 Patient #3 (CMV viremia) seems excluded from the discussion of viral infection in the second paragraph of the discussion.

**Authors’ response: Patient #3 was added to this sentence.**

 The Aspergillus fumigatus case with reads detected below thresholds is interesting and could have more discussion about whether this finding would be communicated to providers for potential followup testing or correlation with other data.

**Authors’ response: Evidence supporting the presence of microorganisms not reaching statistical significance thresholds may be observed. These observations may provide additional value given the clinical context, but with the caveats related to the reduced analytical specificity accompanying them. Such observations can be communicated in the form of a clinical consultation to providers with the associated caveats and understanding that the observations are not part of the CAP-accredited, CLIA-certified, validated test.**

 The presentation in Table 1 is confusing as the MPM is shown as 15 with reference value < 10, seemingly a positive result above threshold, but the text states that it is below threshold.

**Authors’ response:  There are many different comparisons and analyses that are performed in order to determine whether a microbe is “Reported” by Karius. The text stating that the *Aspergillus *cfDNA detected was “below the threshold for a positive test result” refers to a comparison of the amount of *Aspergillus* cfDNA detected in that plasma sample to the amount of *Aspergillus* cfDNA detected in four Negative Control samples that were run on the same batch.  Even though no *Aspergillus *reads were actually detected in these Negative Control Samples, our algorithms act as if a minimal number were for the purpose of performing a statistical comparison.  The number of *Aspergillus* reads detected in that plasma sample were not sufficiently higher than our minimal number to meet the predefined statistical thresholds for reporting (p < 10^-50). We clarified that issue in footnote of Table 1: “ *Aspergillus fumigatus *reads were present in the raw data but below the statistical threshold for a positive test result.”**

**The reference interval is the 97.5% highest MPM value observed in a cohort of 167 plasma samples from healthy blood donors.  Since *Aspergillus *cfDNA is almost never detected in healthy people, the 97.5% highest value is 0.  All quantitative values less than 10 MPM are reported as “< 10 MPM”.  This reference interval value is NOT used in determining whether to report a pathogen, it is provided only as an annotation for Reported organisms.**

 The technical description of the Karius test is fairly general, which is understandable given that it is proprietary, but it does not allow other researchers to assess it independently. More detail could be provided regarding the thresholds for reporting. For example, in the Analysis Pipeline section, the actual quantitation for “high significance” needed to report organisms that are not environmental contaminants as shown by negative controls should be shown. Adequate sequencing depth should be stated as well as the number of base pairs sequenced.

**Authors’ response: As described previously (Blauwkamp et al. Nat Microbiol. 2019 Apr;4(4):663-674), the number of reads required to pass QC is dependent on the total amount of plasma DNA in each sample. Samples that contain higher amounts of cfDNA get more sequencing reads. The number of base pairs sequenced has been highly optimized for the application and remains proprietary.**

 The selection of organisms for reporting and organisms excluded from reporting should be referenced or potentially shown in an appendix.

**Authors’ response:  The full list of organisms for reporting is available online (kariusdx.com/pathogenlist). Please note that all samples were processed with version 3.3 of the Karius Test. The list of organisms specific to version 3.3 can be found here: kariusdx.com/pathogenlist/3.3. For clarification, we added the Karius Test version as well as the link to the pathogen list to the manuscript.**

 The methods state that MPM value is not affected by human cell-free DNA concentration, but others have shown that the human DNA portion does affect the number of reads recovered.

**Authors’ response: The amount of human DNA does indeed affect the number of microbial reads recovered.  That is why we designed our quantitation strategy differently, in a way that is 100% independent of the number of human DNA reads.  See Figure 3d and Supplementary Figure 1 in Blauwkamp et al (Nat Microbiol. 2019 Apr;4(4):663-674) for data addressing performance in light of this issue. There is a 10-fold difference in the amount of human DNA in low versus high, but the MPM quantity is not affected.**

 The authors should state of reference how normalization is done to adjust for samples with differing amounts of human cell-free DNA.

**Authors’ response: As described above, input normalization is not required for accurate quantification. Some additional detail is provided in Blauwkamp et al. Nat Microbiol. 2019 Apr;4(4):663-674.**

# Response

**Published:** 2020-01-15

**Authors:** Steve Miller

**Affiliations:** UCSF, USA

In the article revision, I think the authors responded to my comments, except I would like to see an explanation why case 1 is considered a negative result with 15 MPM where the reference value is < 10, normally a value above the reference range is considered positive.

# Response

**Published:** 2020-01-21

**Authors:** Jose Camargo

**Affiliations:** University of Miami Miller School of Medicine, USA

We would like to apologize for not having clarified this aspect previously. The Karius Test determines presence/absence of a pathogen based on several technical characteristics, such as number of reads aligned and abundance in negative control samples. The reference value based on the asymptomatic/normal population is only provided as annotation and context of what to expect in the "normal population" and is never used in determining whether to filter out or report a pathogen.

# Peer review: Reviewer response for version 2

**DOI:** 10.5256/f1000research.22669.r55948

**Published:** 2019-11-18

**Authors:** Dimitrios Farmakiotis

**Affiliations:** 1 Transplant and Oncology Infectious Diseases, Warren Alpert Medical School, Brown University, Providence, RI, USA

This is an interesting, single-center small case series of the Karius test performance in 10 immunocompromised patients. Agreement with standard microbiological methods was 70% (7 of 10), but with only one "false negative" case of Aspergillus abscess while on treatment. I agree with the authors this is a promising assay that we have all been using in TOID, and as such support its clinical utility, but a scientific manuscript should stick to standardized methodology and report the results unbiased to contribute to our knowledge and set the ground for future studies.

 As such:

1. The authors need to provide more details about the study design. It seems to be a prospective study, but inclusion criteria are not clearly defined. The au mention "availability of kits". Was this a prospective study funded by the sponsor? Were they consecutive pts? More specifically, it seems strange they would enroll a patient who had been on antifungals for so long. Were the tests ordered as part of routine clinical care? The study design needs clarification.
2. Instead of reporting 100% sensitivity "among the patients with HM and confirmed infections", I would prefer to (also) see the "overall agreement" of 70% with standard micro methods.
3. There is limited data on Karius performance for invasive mold infections, and the authors should acknowledge that, referring to additional studies, specifically DMID 2018;92:210 ^1^ (7 of 9 pts correctly identified), Pediatr Blood Cancer 2018;66:e27734 ^2^ (4 of 6 pts correctly identified).

I confirm that I have read this submission and believe that I have an appropriate level of expertise to confirm that it is of an acceptable scientific standard, however I have significant reservations, as outlined above.

## References

[1] : Complexities of Viral Mutation Rates. *Journal of Virology*.2018;92(14) : 10.1128/JVI.01031-17 10.1128/JVI.01031-17 PMC602675629720522

[2] : Temporal trends in the proportion of "cure" in children, adolescents, and young adults diagnosed with chronic myeloid leukemia in England: A population-based study. *Pediatr Blood Cancer*.65(12) : 10.1002/pbc.27422 e27422 10.1002/pbc.27422 30168243

# Response

**Published:** 2019-11-19

**Authors:** Jose Camargo

**Affiliations:** University of Miami Miller School of Medicine, USA

This is an interesting, single-center small case series of the Karius test performance in 10 immunocompromised patients. Agreement with standard microbiological methods was 70% (7 of 10), but with only one "false negative" case of Aspergillus abscess while on treatment. I agree with the authors this is a promising assay that we have all been using in TOID, and as such support its clinical utility, but a scientific manuscript should stick to standardized methodology and report the results unbiased to contribute to our knowledge and set the ground for future studies.

**Authors’ response: **We thank the reviewer for his insightful comments which have improved the quality of the manuscript. You have bought up very valid points. Text has been modified accordingly.  Please find response to your specific comments below.

As such:

1. The authors need to provide more details about the study design. It seems to be a prospective study, but inclusion criteria are not clearly defined. The au mention "availability of kits". Was this a prospective study funded by the sponsor? Were they consecutive pts? More specifically, it seems strange they would enroll a patient who had been on antifungals for so long. Were the tests ordered as part of routine clinical care? The study design needs clarification.

**Authors’ response:** Study design and study subject section have been edited to improve clarity.

2. Instead of reporting 100% sensitivity "among the patients with HM and confirmed infections", I would prefer to (also) see the "overall agreement" of 70% with standard micro methods.

**Authors’ response:** We agree with the reviewer. We have removed this strong statement and abstract and results section have been edited accordingly.

3. There is limited data on Karius performance for invasive mold infections, and the authors should acknowledge that, referring to additional studies, specifically DMID 2018;92:210 ^1^ (7 of 9 pts correctly identified), Pediatr Blood Cancer 2018;66:e27734 ^2^ (4 of 6 pts correctly identified).

**Authors’ response:**  Hong *et al.* and Armstrong *et al.* reports were already included in the discussion.  We have expanded this paragraph in the discussion and added the study by Hill et al.

# Peer review: Reviewer response for version 1

**DOI:** 10.5256/f1000research.21682.r52481

**Published:** 2019-09-04

**Authors:** William Muller

**Affiliations:** 1 Division of Infectious Diseases, Ann & Robert H. Lurie Children’s Hospital of Chicago, Chicago, IL, USA; 2 Northwestern University Feinberg School of Medicine, Chicago, IL, USA

Camargo *et al.* report on results of plasma next-generation sequencing (NGS) for infectious diagnosis in a series of ten patients at risk for infection due to underlying immunocompromise. In addition to demonstrating concordance of NGS testing with conventional microbiologic diagnostic testing methods in seven of the ten patients, NGS testing identified a possibly causative organism in two cases in which conventional test results were negative. In one of the ten patients, PCR and culture detected *Aspergillus*; while NGS was able to detect this organism, it was below the assay detection limit. The authors conclude that this pilot study supports the conclusion that there may be clinical benefit for using this test in this population of patients, warranting more rigorous studies of test performance.

 Overall, the manuscript does not contain significant flaws that would preclude indexing. The authors appropriately acknowledge the limitations of this small pilot study, which by design is not able to determine detailed test performance characteristics such as sensitivity or specificity. Accordingly, my comments are fairly minor and include:

- The methods and results sections report the NGS data in “molecules per microliter” (MPM), which is defined as “the number of DNA sequencing reads from the reported organism present per microliter of plasma.” Although this is a fairly straightforward concept, in the context of sequencing it is not totally clear how the more commonly used concepts of sequencing “breadth” and depth” (e.g., as discussed by Sims *et al.*, 2014 ^1^) apply to MPM. Is it possible to explain MPM in a little more detail? E.g., is there a minimum depth of sequencing that needs to be satisfied for there to be "one" MPM? Does every base in a sequence need to have a certain number of reads?
- The methods also refer to the removal of possible “false-positive calls” from common environmental contaminants – can any examples of organisms that would fit these criteria be provided? Since immunocompromised patients may be at risk of infection from uncommon organisms associated with their environment (particularly uncommon moulds), it is important to understand what may not be reported.
- It’s not completely clear from the text whether results of NGS testing were used for clinical care. Although in most cases it is stated that an established diagnosis of infection was made prior to NGS testing, at least two patients had negative conventional testing and were reported to respond to therapy directed at the organisms identified by NGS. Were these patients responding to empiric treatment, or did NGS results direct the treatment?
- Several places in the manuscript refer to the abdominal infection in the kidney transplant patient as “deep-seated,” but only once is the more medically precise term “abscess” used to describe this infection. Also, can more details about this infection be provided? This is important as the patient appears to have had negative testing (unless the detection limit was lowered below that which the assay typically uses), providing additional clues to situations in which NGS testing may provide false negative results (more on this in the next comment).
- All of the patients had received some antibiotic (antibacterial and/or antifungal) treatment at the time of NGS testing, and one of the more impressive and useful aspects of the test is that it may be able to detect organisms in the setting of effective treatment. However, although a duration of treatment is reported in Table 1, whether this treatment would have been effective against the organism identified is not clear, complicating the interpretation of the data. For example, the Discussion notes that “several months” of treatment had been given to the patient with the *Aspergillus* abscess prior to NGS testing, but it is not totally clear if it was all directed against *Aspergillus* – if so, the negative result is less concerning (and the ability to detect the organism below the reportable threshold is still impressive). Similarly, did patient 10 receive 129 days of antifungal treatment with anti- *Aspergillus* activity prior to his positive test? Although it is understandable why prophylactic treatment would not generally be reported for all patients, was patient 4 receiving any prophylaxis against Pneumocystis?

 

 There are a handful of fairly minor editorial corrections also, including:

- On page 4 the “clinically reportable range” is referred to as “our” clinically reportable range – although it is acknowledged that three of the authors are from the company which performed the NGS testing, given that certain details of the testing are ultimately proprietary perhaps a more generic statement (as simple as “the clinically reportable range”) would be preferred.
- In that same paragraph, although the Mandell textbook is considered by many to be the “go-to” reference for clinical infectious diseases, perhaps it could be referred to as “a clinical infectious diseases reference textbook” or some less subjective phrase? Also, it appears the 8 ^th^ edition is being referenced, which I believe is actually from 2015 (not 2014)?
- The third paragraph in the left column on page 7 contains the phrase “there is limited data…” – as data is plural this should be corrected to “there are limited data…”.

I confirm that I have read this submission and believe that I have an appropriate level of expertise to confirm that it is of an acceptable scientific standard.

## References

[1] : Sequencing depth and coverage: key considerations in genomic analyses. *Nat Rev Genet*.2014;15(2) : 10.1038/nrg3642 121-32 10.1038/nrg3642 24434847

# Response

**Published:** 2019-09-11

**Authors:** Jose Camargo

**Affiliations:** University of Miami Miller School of Medicine, USA

**Reviewer’s comment**:  Camargo *et al.* report on results of plasma next-generation sequencing (NGS) for infectious diagnosis in a series of ten patients at risk for infection due to underlying immunocompromise. In addition to demonstrating concordance of NGS testing with conventional microbiologic diagnostic testing methods in seven of the ten patients, NGS testing identified a possibly causative organism in two cases in which conventional test results were negative. In one of the ten patients, PCR and culture detected *Aspergillus*; while NGS was able to detect this organism, it was below the assay detection limit. The authors conclude that this pilot study supports the conclusion that there may be clinical benefit for using this test in this population of patients, warranting more rigorous studies of test performance.

Overall, the manuscript does not contain significant flaws that would preclude indexing. The authors appropriately acknowledge the limitations of this small pilot study, which by design is not able to determine detailed test performance characteristics such as sensitivity or specificity. Accordingly, my comments are fairly minor and include:

**Author’s response**: Thank you for this thorough review of the manuscript and your insightful comments that have improved the clarity and quality of the paper. Below there is a point-by-point response your inquiries.

**Reviewer’s comment**:

- The methods and results sections report the NGS data in “molecules per microliter” (MPM), which is defined as “the number of DNA sequencing reads from the reported organism present per microliter of plasma.” Although this is a fairly straightforward concept, in the context of sequencing it is not totally clear how the more commonly used concepts of sequencing “breadth” and depth” (e.g., as discussed by Sims *et al.*, 2014 ^1^) apply to MPM. Is it possible to explain MPM in a little more detail? E.g., is there a minimum depth of sequencing that needs to be satisfied for there to be "one" MPM? Does every base in a sequence need to have a certain number of reads?

**Author’s response**: We revised the text to clarify the connection between MPM and sequencing depth. In the Karius Test, the total number of reads observed per organism is often orders of magnitude lower than in applications where the concepts of sequencing breadth and depth are typically used (such as genome assembly). There is indeed a minimum amount of sequencing information that must be obtained in order for a sample to pass quality control criteria which is proportional to the concentration of cell-free DNA in that patient’s sample.  Importantly, the MPM value is not affected by sequencing depth or human cell-free DNA concentration in the sample.

**Reviewer’s comment**:

- The methods also refer to the removal of possible “false-positive calls” from common environmental contaminants – can any examples of organisms that would fit these criteria be provided? Since immunocompromised patients may be at risk of infection from uncommon organisms associated with their environment (particularly uncommon moulds), it is important to understand what may not be reported.

**Author’s response**:  The manuscript was revised to provide further clarity on process to guarantee that the Karius Test does not include frequently observed environmental contaminants. Here, it is important to mention that those taxa are not removed on a case-by-case basis but are completely out of scope for the whole test.

**Reviewer’s comment**:

- It’s not completely clear from the text whether results of NGS testing were used for clinical care. Although in most cases it is stated that an established diagnosis of infection was made prior to NGS testing, at least two patients had negative conventional testing and were reported to respond to therapy directed at the organisms identified by NGS. Were these patients responding to empiric treatment, or did NGS results direct the treatment?

**Author’s response**:  Two aspects of the study should be considered here. i) This was a pilot, proof-of-concept, study in which the majority of patients had an established diagnosis prior to Karius test. ii) We found that NGS correlated well with results from standard diagnostic evaluation. Consequently, the NGS results did not largely influence clinical decision making in this cohort except perhaps in patient #5 where adenovirus PCR in blood was ordered (to confirm DNAemia and monitor viral kinetics which often guide initiation of antiviral therapy) after NGS yielded adenovirus; and in patient #8 in whom clinical presentation was very suggestive of gastrointestinal sepsis due to bacterial translocation in the setting of GVHD, and although blood cultures were negative decision was made to continue empiric antibiotic therapy to complete a course in view of clinical improvement and results of NGS testing.    

**Reviewer’s comment**:

- Several places in the manuscript refer to the abdominal infection in the kidney transplant patient as “deep-seated,” but only once is the more medically precise term “abscess” used to describe this infection. Also, can more details about this infection be provided? This is important as the patient appears to have had negative testing (unless the detection limit was lowered below that which the assay typically uses), providing additional clues to situations in which NGS testing may provide false negative results (more on this in the next comment).

**Author’s response**:  We edited the abstract and manuscript to avoid the term “deep-seated”.  This was a 65-year-old patient who presented with a 1-month history of fever, generalized body aches, malaise and abdominal pain.  Ten months prior, the patient received a kidney transplant complicated with perinephric abscess due to *Aspergillus fumigatus *that required multiple abdominal washouts and several months of antifungal therapy.  Namely, at the time of admission when NGS was sent, the patient had received >6 months of voriconazole (serum levels: 0.5-6.1 mcg/ml; target: 1.5-5 mcg/ml), and micafungin was added for persistent fungal infection. We have added some of this clinical information to the footnote of Table 1. At the time of blood sample for NGS the patient was receiving dual antifungal therapy. We suspect this is the reason why the Karius test was negative. We describe the possibility of false negative results in patients receiving antifungal therapy in the discussion: “prolonged antifungal therapy prior to sample collection (e.g., >7-14 days) might interfere with detection of fungal DNA.”

**Reviewer’s comment**:

- All of the patients had received some antibiotic (antibacterial and/or antifungal) treatment at the time of NGS testing, and one of the more impressive and useful aspects of the test is that it may be able to detect organisms in the setting of effective treatment. However, although a duration of treatment is reported in Table 1, whether this treatment would have been effective against the organism identified is not clear, complicating the interpretation of the data. For example, the Discussion notes that “several months” of treatment had been given to the patient with the *Aspergillus* abscess prior to NGS testing, but it is not totally clear if it was all directed against *Aspergillus* – if so, the negative result is less concerning (and the ability to detect the organism below the reportable threshold is still impressive). Similarly, did patient 10 receive 129 days of antifungal treatment with anti- *Aspergillus* activity prior to his positive test? Although it is understandable why prophylactic treatment would not generally be reported for all patients, was patient 4 receiving any prophylaxis against Pneumocystis?

**Author’s response**: Your point is well taken. The kidney transplant patient received >6 months of anti- *Aspergillus* therapy and was receiving combination of voriconazole plus micafungin at the time of sample collection for NGS testing. Patient 10 had indeed received 129 days of anti-mold prophylaxis/empiric treatment with various agents (including posaconazole, isavuconazole, liposomal amphotericin B [L-AmB]), and was receiving combination of isavuconazole, L-AmB and micafungin at the time of NGS testing. We believe significant fungal burden in the setting of refractory leukemia and prolonged neutropenia facilitated the detection of fungal DNA despite triple antifungal therapy. Patient 4 was not receiving prophylaxis against *Pneumocystis* (atovaquone had been discontinued 3 months prior presentation since CD4>400) but had received 3 days of TMP-SMX treatment dose at the time of NGS testing.  We have edited the discussion to clarify that all patients (except those with viral infections) were on active antimicrobial therapy at the time of NGS testing.

**Reviewer’s comment**:

There are a handful of fairly minor editorial corrections also, including:

 

- On page 4 the “clinically reportable range” is referred to as “our” clinically reportable range – although it is acknowledged that three of the authors are from the company which performed the NGS testing, given that certain details of the testing are ultimately proprietary perhaps a more generic statement (as simple as “the clinically reportable range”) would be preferred.

**Author’s response: **This has been modified.  

**Reviewer’s comment**:

- In that same paragraph, although the Mandell textbook is considered by many to be the “go-to” reference for clinical infectious diseases, perhaps it could be referred to as “a clinical infectious diseases reference textbook” or some less subjective phrase? Also, it appears the 8 ^th^ edition is being referenced, which I believe is actually from 2015 (not 2014)?

**Author’s response: **Text has been modified. 2014 is correct.

**Reviewer’s comment**:

- The third paragraph in the left column on page 7 contains the phrase “there is limited data…” – as data is plural this should be corrected to “there are limited data…”.

**Author’s response: **Thank you. This grammar mistake has been corrected in the revised version.
